# Data set on the experimental investigations of a helical Savonius style VAWT with and without end plates

**DOI:** 10.1016/j.dib.2018.06.113

**Published:** 2018-07-04

**Authors:** T. Micha Premkumar, Seralathan Sivamani, E. Kirthees, V. Hariram, T. Mohan

**Affiliations:** School of Mechanical Sciences, Department of Mechanical Engineering Hindustan Institute of Technology and Science, Chennai, Tamil Nadu, India

**Keywords:** Savonius style, Helical shaped blade, Vertical axis wind turbine, Performance, Low rated wind speeds

## Abstract

The performance test on a helical Savonius style VAWT are carried out with end plates and without end plates for low wind velocities from 3 m/s to 6 m/s. The raw data measured using instruments are recorded using digital acquisition system. These data are processed and presented as dimensionless parameters namely, coefficient of power, coefficient of torque and tip speed ratio in order to compare it with other VAWTs.

**Specifications Table**

**Table:** 

Subject area	*Renewable energy*
More specific subject area	*Wind engineering*
Type of data	*Figures and Tables*
How data was acquired	*Experimental investigations* of a helical Savonius style VAWT with and without end plates *using cup type anemometer, torque sensor, RPM sensor by mechanically loading it with a dynamometer and recording the data using data acquisition system*
Data format	*Raw, processed, calculated, tabulated, plotted, analyzed*
Experimental factors	*Data are normalized as per wind turbine study standards*
Experimental features	*Helical Savonius style VAWT with and without end plates working on the principle of drag is tested for wind speeds ranging from 3 m/s to 6 m/s*
Data source location	*Mechanical Engineering Department, Hindustan Institute of Technology and Science, Chennai, Tamilnadu, India*
Data accessibility	*Data on the performance studies are incorporated in this article*

**Value of the data**

• Data set on helical Savonius style VAWT with end plates and without end plates at low wind velocities provide an insight to understand its aerodynamic performance behavior.
• Data set would give researchers to design a packaged installation of helical Savonius style VAWT on roof top of urban home clusters to generate power.
• Data set to provide a benchmark for future simulation studies on this type of VAWT and possible aerodynamic design improvements.

## 1 Data

**Fig. 1:**
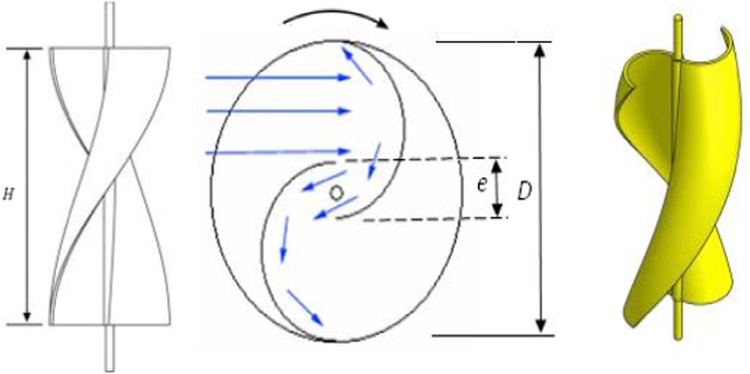
Typical helical Savonius style VAWT.

Savonius style vertical axis wind turbine (VAWT) is the simplest among all the modern types of wind energy conversion systems. With its self starting ability, it can operate at relatively low velocity winds and irrespective of the direction of wind, the rotor shaft of VAWT rotate such a way that convex side of the blade heads into the wind. The wind force is larger on the cupped side than the rounded side. This wind which curves around the cupped side exert a reduced pressure and this help to drive the rotation. As can be seen in Fig. 1, the wide gap between the two inner edges of the half cylinder allows the air to whip out the air in forward moving cupped face so that the fresh air is allowed to hit the blade.

Earlier, researchers conducted studies to understand the aerodynamic characteristic behaviors and influence of geometric design parameter on Savonius VAWT [1], [2]. Recently, it was established that helical Savonius VAWT had a better performance with positive static torque coefficient at all rotor angles [3], [4], [5]. Study by researchers also showed that end plates increased the aerodynamic performance of Savonius VAWT [6]. Therefore, the focus of this present data set is on the effect of end plates on improving the aerodynamic performances of the helical Savonius style rotor system as represented in Tables 2 and 3. The aerodynamics performance of the helical Savonius VAWT is represented in terms of dimensionless parameters namely, coefficient of power (*C*_P_), coefficient of torque (*C*_T_) and tip speed ratio (*λ*).The data set reported here (refer Tables 2 and 3) is based on the study carried out at low wind velocities ranging from 3 m/s to 6 m/s.

**Table 1:** Dimensional details of helical Savonius VAWT.

Wind speed (*V*)	3–6 m/s
Aspect ratio(*A*_R_=*H*/*D*)	1.50
Swept area (*A*)	0.666 m^2^
Diameter of the VAWT (*D*)	0.666 m
Height of the VAWT (*H*)	1 m
Overlap ratio (*δ*=*e*/*d*)	0.147
Twist angle	0–180°
Overlap distance (*e*)	52.83 mm
Blade chord length $\left(d=333+\frac{e}{2}\right)$	359.417 mm
Number of blades	Two
Type	Helical Savonius
Solidity	2.16
End plate diameter (*D*_o_)	799 mm

**Table 2:** Performance characteristics of helical Savonius VAWT at various wind velocity without endplates.

**Wind velocity (*V***_**average**_**) (m/s)**	**Torque (*T*) N/m**	**Rotor shaft speed of VAWT (*N*) RPM**	**Tip speed ratio (*λ*)**	**Coefficient of torque (*C***_**T**_**)**	**Coefficient of power (*C***_**P**_**)**
3.385	0.16575	24.5095	0.25236	0.252790	0.0268
3.521	0.21572	26.0449	0.25785	0.216041	0.0330
4.097	0.20254	46.2138	0.40167	0.227520	0.0356
4.264	0.25331	47.9288	0.31003	0.179218	0.0356
4.589	0.30245	62.7076	0.47623	0.153274	0.0365
4.608	0.31435	85.3128	0.64536	0.149825	0.0404
5.177	0.41466	87.6077	0.45519	0.147622	0.0419

**Table 3:** Performance characteristics of helical Savonius VAWT at various wind velocity with endplates.

**Wind velocity (*V***_**average**_**) (m/s)**	**Torque (*T*) N/m**	**Rotor shaft speed of VAWT (*N*) RPM**	**Tip speed ratio (*λ*)**	**Coefficient of torque (*C***_**T**_**)**	**Coefficient of power (*C***_**P**_**)**
3.7572	0.18357	44.304	0.4110	0.1913	0.0393
4.3093	0.23734	63.669	0.5150	0.1880	0.0484
4.3263	0.28076	79.046	0.6368	0.1421	0.0453
4.5775	0.31201	81.705	0.7460	0.1699	0.0464
4.9248	0.40913	91.663	0.8487	0.1268	0.0512
5.1293	0.47684	99.663	0.8685	0.1148	0.0540

## 2 Experimental design, materials and methods

### 2.1 Wind turbine design and experimental setup

**Fig. 2:**
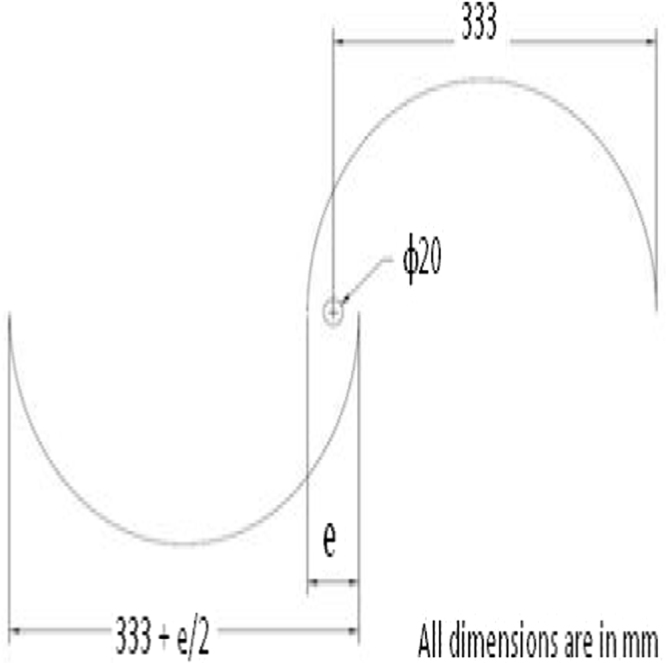
Schematic dimensional detail of the helical Savonius rotor.

Saha and Sukanta Roy [7] reported that an aspect ratio (*A*_R_=*H*/*D*) of 1.50 gives a good performance characteristic. Hence, this aspect ratio (*A*_R_) of 1.50 is chosen in the design of helical Savonius VAWT. The height of the helical Savonius VAWT (*H*) is kept as 1 m. Therefore, the swept diameter of this VAWT (*D*) is 0.666 m. Bhaumik and Gupta [8] proved that the optimum overlap ratio (*δ*=*e*/*d*) is 0.147 for the helical Savonius rotor. The schematic dimensional detail of the helical Savonius rotor blade is shown Fig. 2 and the dimensional details are listed in Table 1.

**Fig. 3:**
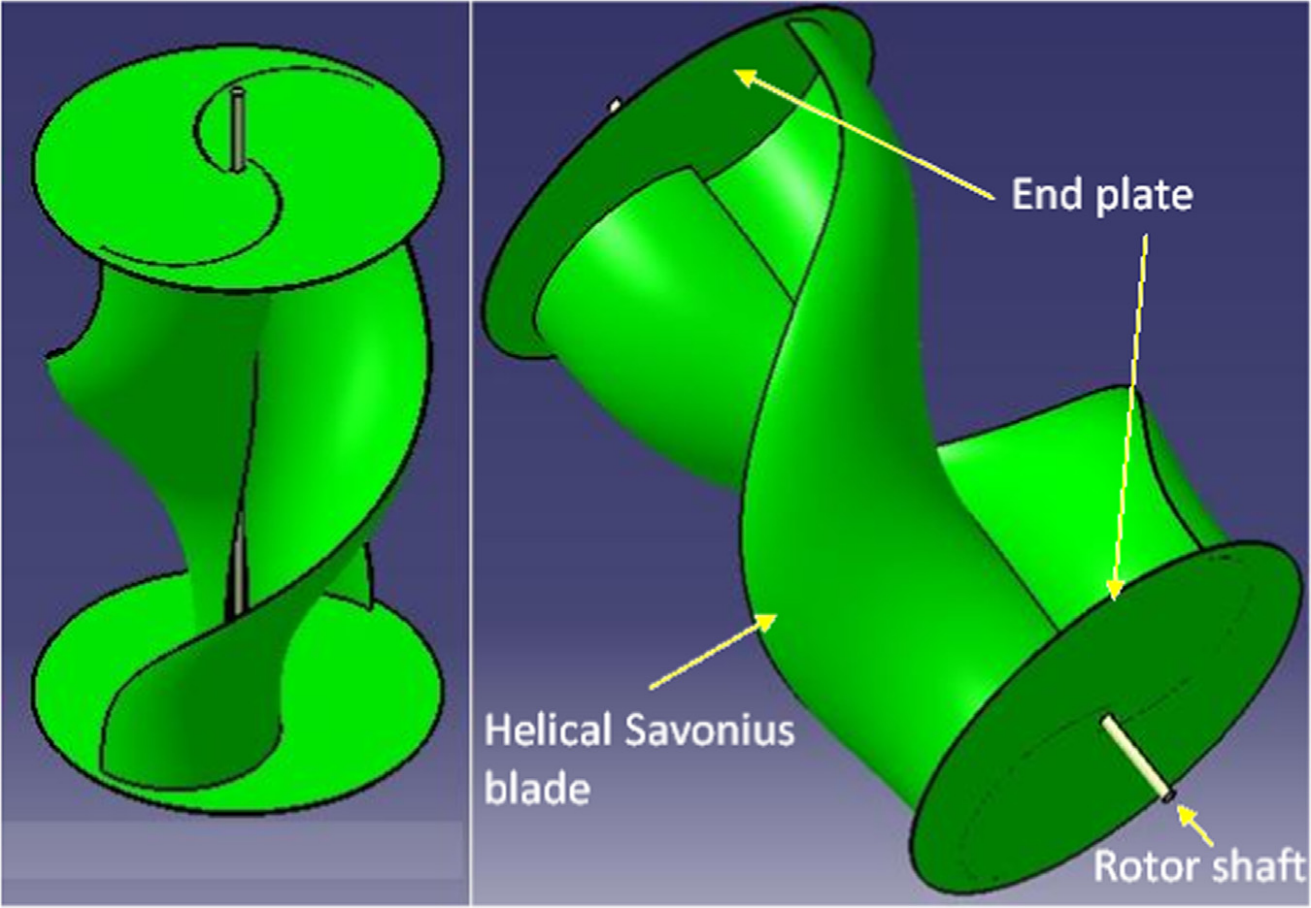
3D model of the helical Savonius blade with end plates.

Fig. 3 shows the 3D model of the helical Savonius style VAWT with end plates as per the dimensions mentioned in Table 1. The end plates are kept 20% larger than the overall diameter (*D*) with a dimension of 799 mm. The blades are twisted up to 180° from top to bottom and the twist angle of the blade can also be seen in the Figure.

**Fig. 4:**
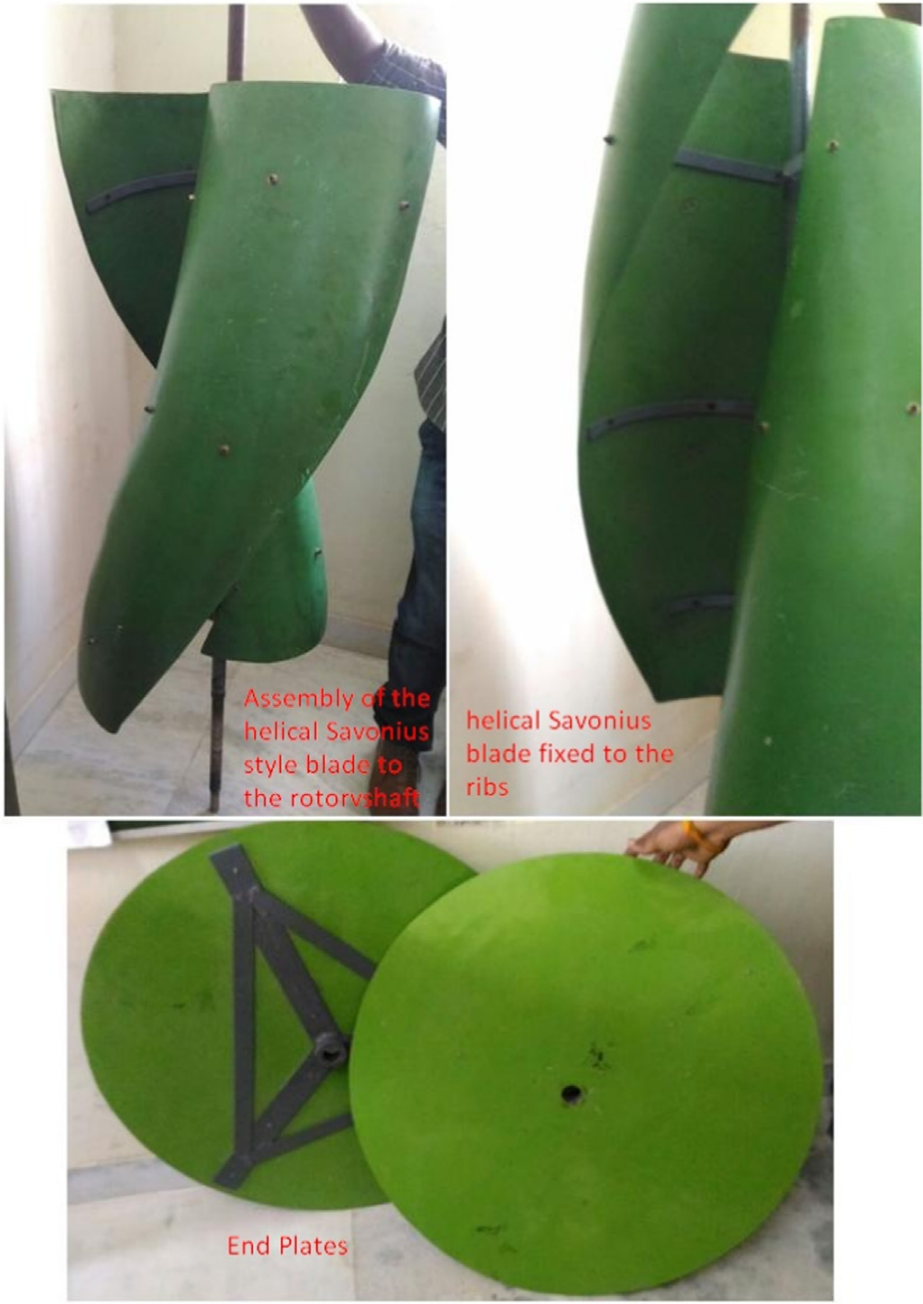
Assembled helical Savonius style blade to the rotor shaft of VAWT.

The helical Savonius blades as well as the end plates are manufactured using fiber reinforced plastic material (FRP). The FRP blades are connected to the rotor shaft by using three pairs of flat semicircular metal ribs. The assembled helical Savonius blade with the rotor shaft is shown in Fig. 4. Taper roller bearing and thrust ball bearing are used at both the ends for mounting the helical Savonius VAWT on the experimental test rig. The taper roller bearing is fixed in the housing and the thrust ball bearing is fixed above to it. The radial load acts on the taper roller bearing and the axial load is taken care by the thrust ball bearing.

**Fig. 5:**
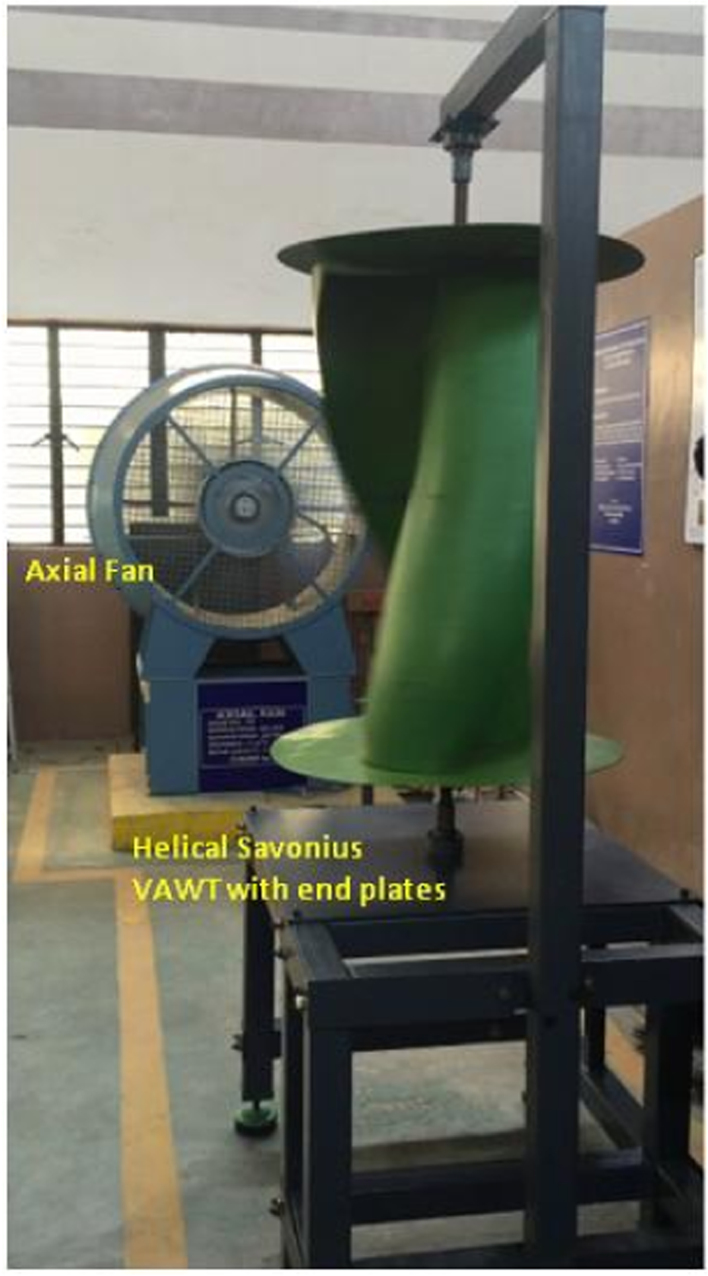
Experimental test rig with helical Savonius VAWT.

Fig. 5 shows the helical Savonius VAWT on the experimental test rig used for this present study. An axial fan with a variable frequency drive (ABB^TM^ make) is used to generate free stream of air with its velocity varying from 3 m/s to 6 m/s. Cup type anemometer is used to measure the wind speed (*V*). A torque sensor (Sushma™ make) is used to measure the torque of this rotating system (*T*). A non-contact type photo electric sensor is used for measuring the rotational speed of the VAWT (*N*). All the measured data is recorded in a computer using data acquisition system powered by NI instruments with Labview software. With these data, the tip speed ratio, coefficient of power (C_p_) and coefficient of torque (*C*_T_) are calculated. The error of these calculated parameters are estimated to be ±2.24% which is based on the standard of error estimation [9]. The performance test is conducted by loading the helical Savonius VAWT using a brake drum type dynamometer. 1 mm thick fishing nylon type thread is wound over the groove of the drum which is fixed to the rotor shaft of the helical Savonius VAWT. One end of this nylon thread is kept fixed and to its other end, a weighing pan is attached. The performance test is carried out by varying the load on the weighing pan from no load to maximum load for different wind velocities.

### 2.2 Performance and loading test data

The performance index of a typical VAWT is expressed in terms of coefficient of power (*C*_p_) and coefficient of torque (*C*_T_).Theoretical available power is expressed as $P_{\mathrm{available}}=\frac{1}{2}\rho Av^{3}$where *A* is the swept area (m^2^) and *V* is the velocity of the wind (m/s).

Power available at the rotor shaft can be expressed as

$$P_{\mathrm{rotor}\;\mathrm{shaft}}=\frac{2\pi NT}{60}$$

where *T* is the brake torque produced (N m) and *N* is rotational speed of the rotor shaft (rpm)Tip speed ratio (*λ*) is expressed as $TSR=(\omega \times R)/V=\;(\frac{2\pi N}{60})\times R)/V$where ω is the rotor rotational speed in terms of radians/second and R is the rotor radius in metre.Coefficient of performance (*C*_p_) is given as $\;C_{p}=\frac{P_{rotor\;shaft}}{P_{available}}$Coefficient of the torque (*C*_T_) is mentioned as $\;C_{T}=\frac{T}{\frac{1}{2}\rho AV^{2}R}=\frac{F\times r_{p}}{\frac{1}{2}\rho AV^{2}R}$

Tables 2 and 3 lists the performance characteristics of a helical Savonius VAWT at various wind velocity without end plate and with end plates respectively. On comparing the performance parameters namely, *C*_P_ and *C*_T_ of both helical Savonius VAWT without end plate and with end plate, the performance of helical Savonius VAWT with end plate is observed to be better. Also, the *C*_P_ of helical Savonius style wind turbine with end plate is observed to be nearly even.

**Fig. 6:**
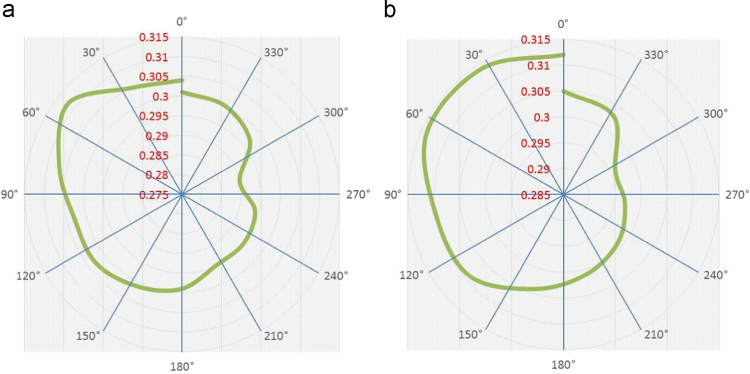
Variations of angle Vs torque for a helical Savonius style VAWT (a) without end plates and (b) without end plates.

Polar plots for angle Vs torque for a helical Savonius style VAWT with end plates and without end plates are plotted in order to analyze the distribution of torque at various rotational angles of the VAWT׳s blade in its 360° of rotation. Fig. 6(a) and (b) shows the variations of torque with respect to angle in its rotational direction for helical Savonius style VAWT without end plates and with end plates respectively for a wind velocity of 4.5 m/s. Highest torque value of 0.310 is observed at 51° for helical Savonius style VAWT without end plates and its values are found to better between 30° to 60° angle of rotation. Similarly, the highest torque value of 0.314 is reported at 43° angle for helical Savonius style VAWT with end plates. In general, for a single revolution of VAWT, the torque is initially high (0° to 150° angle) and later it decreases gradually.
